# Marital Support Reciprocity and Life Satisfaction Among Older Koreans

**DOI:** 10.1093/geroni/igab046.3310

**Published:** 2021-12-17

**Authors:** Hye Soo Lee

**Affiliations:** Oregon State University, Corvallis, Oregon, United States

## Abstract

The importance of reciprocity in social support for well-being has been shown, but few studies have investigated marital support reciprocity in older Korean samples. This study examined the associations between three types of marital support reciprocity and life satisfaction, stratified by age and gender. The sample consisted of 1,578 men and 1,464 women from the 2017 National Survey of Older Koreans, divided into young-old (65-74) and old-old (75+) groups (M age = 75.06, SD = 6.35). Participants self-reported emotional, instrumental, and physical support provided to and received from spouses, and life satisfaction (LS). Regression models controlling for covariates showed that results varied by age and gender. For young-old males, received emotional and provided instrumental support were positively associated with LS. For young-old females, both received and provided emotional support, and received instrumental support, were positively associated with LS, but provided physical support showed negative associations. For old-old males, providing emotional support was positively associated with LS; for old-old females, only received emotional support was significant. Using interaction terms to assess reciprocity, young-old females and old-old males showed reciprocity effects for instrumental support. When participants provided and received high levels of support, life satisfaction levels were high. However, when participants provided low levels of support, received support was not significant. Thus, the effects of receipt and provision of support on LS varied by age and gender among older Koreans, but reciprocity of instrumental support was only important for young-old women and old-old men.

